# Assessment of Physical and Mechanical Properties of Auto-Polymerized Acrylic Resins Using a Custom Pressure Vessel Technique: An In Vitro Study

**DOI:** 10.7759/cureus.96880

**Published:** 2025-11-15

**Authors:** Yugandhar Garlapati, B Rama Mohan Reddy, Poornima Nayak, Jashva V Kogila, C Venkata Reddy, K N Anand Kumar, P Sindhu Chandrika, Shivukumar Poojary, Seema Gupta

**Affiliations:** 1 Department of Orthodontics and Dentofacial Orthopedics, Government Dental College and Hospital, Kadapa, IND; 2 Department of Orthodontics and Dentofacial Orthopedics, Kothiwal Dental College and Research Centre, Moradabad, IND

**Keywords:** curing, hardness, polymethyl methacrylate, pressure, staining

## Abstract

Introduction: Auto-polymerized acrylic resins are essential in dentistry because of their versatility; however, their suboptimal physical and mechanical properties can limit their clinical performance. This study aimed to evaluate the impact of custom-made pressure vessel treatment on the physical, mechanical, and aesthetic properties of these resins, specifically by comparing polymerization shrinkage, assessing flexural strength, measuring water sorption, evaluating surface hardness, analyzing porosity, and determining color stability after staining.

Materials and methods: This in vitro study involved 30 specimens of auto-polymerized acrylic resin, equally divided into a control group (n = 15, cured under ambient conditions) and an experimental group (n = 15, cured in a custom pressure vessel at 2.2-3.0 bar). Specimens were prepared in standardized molds, polished, and stored in distilled water to mimic oral conditions. The outcomes assessed included polymerization shrinkage (volumetric analysis), water sorption (weight change post-immersion), flexural strength (three-point bending test), surface hardness (Vickers test), porosity (scanning electron microscopy), and color stability (Commission Internationale de l'Éclairage (CIE) L*a*b* parameters post-coffee staining). All measurements used calibrated instruments, and data were analyzed using independent t-tests (p < 0.05) after confirming normality using the Shapiro-Wilk test.

Results: The pressure-treated group exhibited significantly reduced water sorption (2.30 ± 1.11%) compared to the control group (3.47 ± 1.48%), with a mean difference of 1.17% (p = 0.021), and increased flexural strength (66.62 ± 8.67 MPa) compared to the control group (58.64 ± 10.97 MPa, p = 0.035), indicating improved material density and mechanical durability. No significant differences were found in polymerization shrinkage (p = 0.273), surface hardness (p = 0.840), or color stability, suggesting that the pressure treatment maintained the dimensional and esthetic integrity.

Conclusion: Custom pressure vessel treatment enhanced the water sorption resistance and flexural strength of auto-polymerized acrylic resins without affecting their shrinkage, hardness, or color stability. These findings suggest the potential for improved durability in dental appliances, supporting the adoption of this technique in clinical practice, pending further in vivo validation.

## Introduction

Auto-polymerized acrylic resins, also known as cold-cure or self-cure resins, are indispensable materials for the fabrication of removable appliances, retainers, splints, and functional devices, such as activators and expanders. Their popularity stems from several advantages, including ease of manipulation at the chairside, rapid polymerization without the need for heat, affordability, and satisfactory esthetic properties that blend well with natural dentition [1,2]. These resins consist primarily of polymethyl methacrylate (PMMA) and undergo chemical initiation via tertiary amines, allowing quick setting under ambient conditions [2]. However, despite their widespread use, auto-polymerized resins exhibit inherent limitations that compromise their clinical performance. Polymerization shrinkage, typically ranging from 3 to 7%, arises from the conversion of monomers to polymers, leading to dimensional instability, internal stress, and poor adaptation to dental structures [3]. Additionally, incomplete polymerization often results in high porosity due to entrapped air or monomer evaporation, which weakens the flexural strength of the material, increases water sorption, and reduces surface hardness [4]. Excessive water sorption can cause swelling, discoloration, and microbial colonization, whereas low flexural strength increases the risk of fracture under occlusal loads, potentially leading to appliance failure and patient discomfort [5]. These drawbacks not only shorten the longevity of orthodontic appliances but also affect treatment outcomes, such as retention stability in retainers or force delivery in functional appliances.

To mitigate these issues, researchers have explored various enhancements, including the incorporation of reinforcing fibers, alternative initiators, and modified curing protocols [6]. Heat polymerization, which is superior in reducing porosity and improving mechanical properties, requires specialized equipment, such as water baths and extended processing times (1.5-2 hours), making it impractical for routine orthodontic applications where immediacy is key [2,3]. Pressure-assisted polymerization is a promising alternative because it compresses the resin matrix during curing to minimize voids and enhance density. Studies on heat-cured resins have demonstrated that high pressures of approximately 500 megapascals (MPa) reduce the shrinkage and porosity by limiting bubble formation and promoting uniform monomer conversion [7]. However, there is a notable gap in the literature regarding the application of custom-made, low-cost pressure vessels specifically for auto-polymerized resins in orthodontic contexts, where cost-effectiveness and simplicity are paramount.

The aim of this study was to evaluate the impact of custom-made pressure vessel treatment on the physical and mechanical properties of auto-polymerized acrylic resins. The specific objectives were to compare polymerization shrinkage between pressure-treated and untreated groups using volumetric measurements, assess differences in flexural strength via three-point bending tests, measure water sorption through weight changes after immersion, evaluate surface hardness using Vickers hardness testing, analyze porosity via scanning electron microscopy (SEM), and determine color stability using reflectance spectrophotometry after staining.

## Materials and methods

This in vitro experimental study was conducted in a controlled laboratory setting within the Department of Orthodontics at the Government Dental College and Hospital, Kadapa, Andhra Pradesh, India. The study duration was extended over a period of approximately six months, from January 2023 to June 2023. As this was an in vitro investigation involving no human participants, animal subjects, or biological tissues, ethical approval from an institutional review board was not required in accordance with guidelines from the Indian Council of Medical Research for non-clinical material-based studies, although all procedures adhered to standard laboratory safety protocols to prevent material wastage or environmental hazards.

A priori sample size calculation was performed using G*Power software (version 3.1.9.7; Heinrich-Heine-Universität Düsseldorf, Düsseldorf, Germany). The calculation was based on an effect size of 1.12, which was obtained from a study by Kostić et al. [8] that investigated water sorption in different acrylic materials. The analysis indicated that a minimum sample size of 15 specimens per group (a total of 30 for two groups) was sufficient to achieve a statistical power of 90% at a significance level (alpha) of 5%.

**Figure 1:**
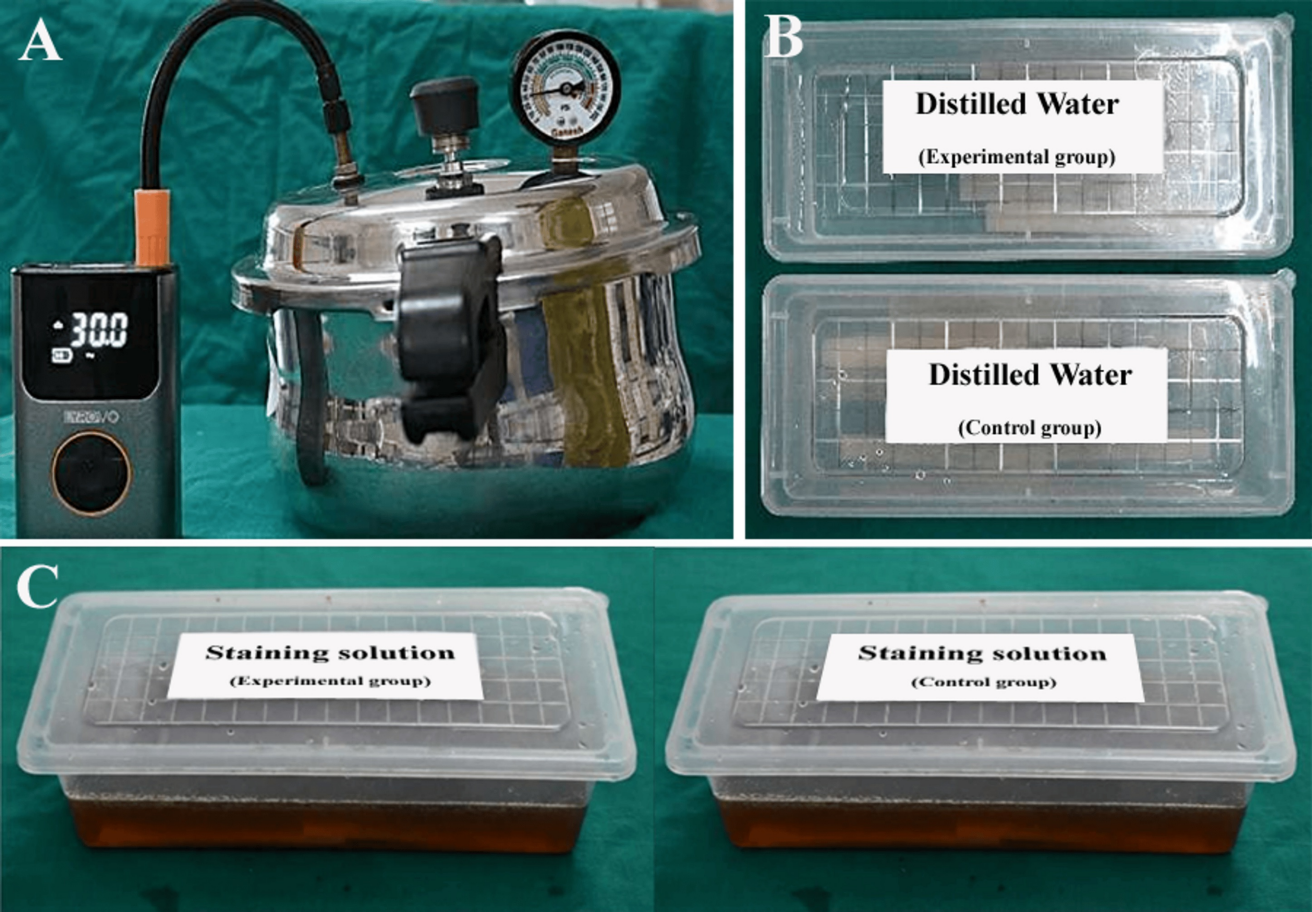
(A) Custom pressure vessel filled with lukewarm distilled water and pressurized to 2.2-3.0 bar for polymerization of the experimental group, (B) Control and experimental group specimens stored in distilled water at 37 °C for 24 hours, and (C) Control and experimental group specimens immersed in coffee staining solution for seven days at 37 °C to check for color stability. Original images of samples used in the study.

To achieve standardization across all specimens, a custom-made metal mold was used with fixed dimensions of 125 mm in length, 12.7 mm in width, and 3.2 mm in thickness, featuring a precisely machined rectangular cavity to ensure uniform shape and volume, with all molds inspected for defects prior to use and cleaned with isopropyl alcohol between batches to avoid contamination. The detailed procedure commenced with specimen fabrication using cold-cure auto-polymerized acrylic resin (DPI RR, Dental Products of India, Mumbai, India), in which the polymer powder and monomer liquid were mixed in a clean porcelain jar at room temperature (25 ± 2 ^0^C) following the manufacturer's specified ratio of 3:1 by volume or 2.5:1 by weight, with the monomer poured first and the polymer added gradually over 30 s while stirring with a spatula to minimize air bubbles. Mixing was continued until the dough stage was reached (4-5 minutes), identified by a smooth, elastic, non-sticky texture, in a dust-free environment with no air currents from fans or open windows to prevent monomer evaporation or contamination. The dough was packed into a pre-lubricated metal mold using hand pressure or a spatula to ensure even distribution without overpacking (which could cause flash) or underpacking (which could create voids), followed by placement of a glass slab over the mold for uniform compression and surface flattening under consistent manual force for 1 min (Figure 1).

For the control group (n = 15, untreated group), the molds were left to polymerize at ambient room temperature and atmospheric pressure for 15-20 minutes until set. For the experimental group (n = 15, pressure-treated group), molds were immediately transferred to a custom-made pressure vessel (fabricated from stainless steel with a pressure gauge and valve, no specific commercial brand) filled with lukewarm distilled water (40-46 ^0^C) to 3-5 cm below the rim, and then pressurized to 2.2-3.0 bar using a standard tire inflator connected via a hose, maintaining the pressure for 15-20 minutes to facilitate uniform hydrostatic transmission through the water medium, which compressed the resin matrix without direct heat application (Figure 1A). After polymerization, the specimens were demolded, edges trimmed with a tungsten carbide bur, and surfaces polished progressively using 400-1200 grit silicon carbide papers followed by pumice slurry on a lathe at 2000 rpm for two minutes per side to achieve a standardized smooth finish. They were then rinsed and stored in distilled water at 37 ^0^C for 24 h to simulate oral conditions and allow for any residual monomer leaching (Figure 1B).

Calibration of measurement instruments was conducted before each testing session; the digital caliper for dimensional assessments was calibrated against certified gauge blocks to an accuracy of 0.01 mm, the digital pocket scale (ATOM Selves-MH 200 GM, Zhejiang Junkaishun Industrial & Trade Co. Ltd., Zhejiang, China) was zeroed and verified with standard weights ranging from 1 g to 200 g, achieving a precision of ±0.01 g, while the universal testing machine was calibrated using known load cells, and the Vickers microhardness tester was calibrated with reference hardness blocks to ensure indentations within 1% variability. Reliability was established through pilot testing on five additional specimens, where intra-examiner reliability for measurements such as hardness and shrinkage was assessed via the intraclass correlation coefficient (ICC > 0.95). All primary assessments were performed by a single trained investigator who repeated 20% of the tests blindly after a one-week interval to confirm consistency, yielding no significant differences (p > 0.05) and thus affirming high reproducibility.

Outcome assessment involved multifaceted evaluations starting with polymerization shrinkage, measured volumetrically by recording initial mold dimensions (V0 = length × width × height) using a calibrated digital caliper and final specimen dimensions (Vf) immediately after demolding and polishing. The percentage shrinkage was calculated as:

\begin{equation} \text{Shrinkage (%)} = \frac{V_0 - V_f}{V_0} \times 100 \end{equation}

The three measurements per dimension for precision were then averaged. Water sorption was determined by drying the specimens with absorbent paper, obtaining the initial weight (m1) on the digital pocket scale, immersing them in 100 mL distilled water at 37 ^0^C for 24 h, then blotting dry and reweighing (m2), and repeated twice per specimen for reliability.

\begin{equation} \text{Water sorption (%)} = \frac{m_2 - m_1}{m_1} \times 100 \end{equation}

**Figure 2:**
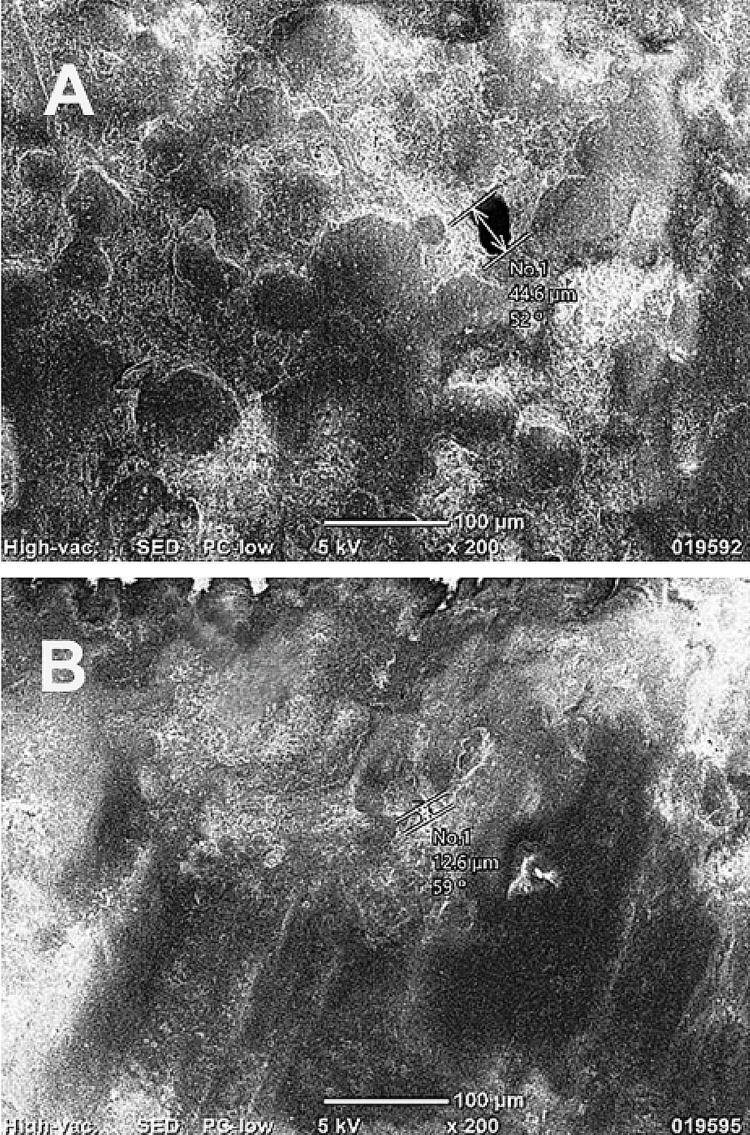
Scanning electron microscopy (SEM) images showing porosity in control (A) and experimental (B) acrylic models. The control specimen (A) demonstrated larger and irregular pores, whereas the pressure-treated specimen (B) exhibited reduced porosity and a more homogeneous matrix, indicating improved polymerization density under pressure-curing conditions (magnification ×1000). Original SEM images of samples from the study.

Flexural strength was evaluated via a three-point bending test on a universal testing machine at 37 ^0^C, with specimens positioned on supports 50 mm apart and loaded at the midpoint with a crosshead speed of 5 mm/min until fracture. The maximum load was recorded in MPa, with machine calibration ensuring <1% error in force application. The surface hardness was assessed using the Vickers hardness test on a microhardness tester with a diamond indenter by applying a 100 g load for 15 s at five random points per specimen (avoiding edges). The average Vickers hardness value (HV) was calculated from indentation diagonals measured under 40x magnification, with tester reliability confirmed by standard blocks yielding consistent readings. Porosity was analyzed through SEM on selected sectioned specimens (cut transversely with a diamond disc), cleaned ultrasonically in distilled water for five minutes, air-dried, sputter-coated with gold-palladium for conductivity, and imaged at 1000x magnification to qualitatively compare the pore size, distribution, and matrix homogeneity between groups (Figure 2).

In the color stability study, a staining solution was prepared with 2.8 g Nescafé coffee powder (Nestlé S.A., Vevey, Switzerland) dissolved in 500 mL distilled water, and 15 specimens per group were immersed for seven days at 37 ^0^C (Figure 1C). Color parameters were assessed before and after immersion using a reflectance spectrophotometer under D65 illumination by measuring L* (lightness, ranging from 0 for black to 100 for white), a* (red-green axis, with positive values for red/magenta, negative for green, and zero for neutral), and b* (yellow-blue axis, with positive values for yellow, negative for blue, and zero for neutral) in the Commission Internationale de l'Éclairage (CIE) Lab* color space.

Here is the formula for total color difference (ΔE):

\begin{equation} \Delta E = \sqrt{(\Delta L^*)^2 + (\Delta a^*)^2 + (\Delta b^*)^2} \end{equation}

where ΔL*, Δa*, and Δb* are the differences in the L*, a*, and b* values before and after staining, respectively. The instrument was calibrated against a white standard tile for accuracy, data were collected blindly to group assignment, and outliers were checked using Grubbs' test to ensure a reliable evaluation of the treatment's effects.

Data were entered into Microsoft Excel and analyzed using IBM SPSS Statistics for Windows, Version 25 (Released 2017; IBM Corp., Armonk, New York, United States). The Shapiro-Wilk test confirmed the normality of the data. An independent samples t-test was used to compare physical and aesthetic properties between the study groups, with a p-value of < 0.05 considered statistically significant.

## Results

The Shapiro-Wilk test results indicate that all parameters in both the control and experimental groups have p-values > 0.05. This confirms that the data for all measured variables are normally distributed, validating the use of parametric statistical tests for subsequent analyses (Table 1).

**Table 1:** Analysis of distribution of data with the Shapiro-Wilk test. MPa: Megapascals; HV: Vickers hardness value; water sorption and polymerization shrinkage are measured as mean change in percentage (%); RG: Red-blue color shift; YB: Yellow-blue color shift. *p < 0.05 denotes statistical significance using the Shapiro-Wilk test

Parameters	Control (n = 15, untreated group)		Experimental (n = 15, pressure-treated group)	
	Test value	p-value	Test value	p-value
Water sorption (%)	0.95	0.576	0.24	0.282
Polymerization shrinkage (%)	0.98	0.949	0.94	0.346
Flexural strength using the three-point bending test (MPa)	0.95	0.498	0.18	0.179
Surface hardness using the Vickers hardness test (HV)	0.97	0.885	0.92	0.174
Lightness (L*)	0.78	0.530	0.77	0.450
RG color shifts (a*)	0.99	0.991	0.95	0.743
YB color shifts (b*)	0.90	0.424	0.88	0.318
Total color difference (ΔE)	0.99	0.981	0.95	0.629

Comparative analysis of physical properties between the control and experimental groups revealed statistically significant differences in the two key parameters. Regarding water sorption, the experimental group cured under pressure demonstrated a significantly lower value (2.30 ± 1.11%) compared to the control group cured at atmospheric pressure (3.47 ± 1.48%), with a mean difference of 1.17% (p = 0.021). This indicates that the pressure-curing method effectively reduced the water sorption of the auto-polymerized acrylic resin. Furthermore, the experimental group exhibited a significantly higher flexural strength (66.62 ± 8.67 MPa) than the control group (58.64 ± 10.97 MPa), with a mean difference of -7.98 MPa (p = 0.035), suggesting a substantial improvement in flexural strength. In contrast, the differences in polymerization shrinkage (Control: 3.06 ± 2.80% vs. Experimental: 2.05 ± 2.10; p = 0.273%) and Vickers hardness (Control: 16.0 ± 1.64 HV vs. Experimental: 16.1 ± 0.98 HV; p = 0.840) were not statistically significant. The conclusion drawn is that curing auto-polymerized acrylic resin in a custom pressure vessel significantly enhances its resistance to water sorption and improves its flexural properties, thereby potentially increasing the durability and clinical performance of the material without significantly altering its polymerization shrinkage or surface hardness under the conditions of this study (Table 2).

**Table 2:** Comparison of physical properties of study groups using an independent t-test. MPa: Megapascals; HV: Vickers hardness value; water sorption and polymerization shrinkage are measured as mean change in percentage (%). *p < 0.05 denotes statistical significance using an independent sample t-test.

Parameters	Control (n = 15, untreated group)	Experimental (n = 15, pressure-treated group)	Mean difference	T stats	p-value
Water sorption (%)	3.47 + 1.48	2.30 + 1.11	1.17	2.44	0.021*
Polymerization shrinkage (%)	3.06 + 2.80	2.05 + 2.10	1.01	1.11	0.273
Flexural strength using the three-point bending test (MPa)	58.64 + 10.97	66.62 + 8.67	-7.98	2.21	0.035*
Surface hardness using the Vickers hardness test (HV)	16.0 + 1.64	16.1 + 0.98	-0.10	0.20	0.840

Analysis of color stability revealed no statistically significant differences between the control and experimental groups across all measured parameters. The L* value was comparable between the control (50.75 ± 2.94) and pressure-cured groups (49.29 ± 2.06), with a non-significant mean difference of 1.46 (p = 0.126). Similarly, the red-green (RG) and yellow-blue (YB) color shifts showed negligible and non-significant variations. Consequently, the ΔE was not significantly different between the groups (p = 0.274). The inference drawn from these results is that the pressure-curing protocol, which is beneficial for the mechanical and physical properties as previously determined, does not exert a significant effect on the color stability or optical properties of the auto-polymerized acrylic resin. This indicates that the intervention improved the performance of the material without compromising its esthetic characteristics (Table 3).

**Table 3:** Comparison of color stability of study groups using an independent t-test. RG: Red-blue color shift; YB: Yellow-blue color shift. p > 0.05 denotes no statistical significance using an independent samples t-test.

Variables	Control (n = 15, untreated group)	Experimental (n = 15, pressure-treated group)	Mean difference	T stats	p-value
Lightness (L*)	50.75 + 2.94	49.29 + 2.06	1.46	1.57	0.126
RG color shifts (a*)	1.66 + 0.52	1.61 + 0.43	0.05	0.28	0.776
YB color shifts (b*)	6.04 + 0.93	6.09 + 0.98	-0.05	0.14	0.887
Total color difference (ΔE)	0.50 + 0.17	0.43 + 0.14	0.07	1.23	0.274

## Discussion

The findings of this in vitro study underscore the potential of custom-made pressure vessel treatment during polymerization to enhance the selection of the physical and mechanical properties of auto-polymerized acrylic resins, without compromising esthetic attributes. By applying hydrostatic pressure (2.2-3.0 bar) in a water medium, the process appears to optimize resin matrix formation, primarily by mitigating internal voids and improving molecular packing, which aligns with the fundamental principles of polymer science, where external pressure can counteract volumetric contraction during free radical polymerization [7]. This selective improvement is particularly noteworthy in the context of dental applications, where auto-polymerized acrylics are favored for their chairside convenience in fabricating orthodontic appliances, denture bases, and provisional restorations; however, they are often criticized for suboptimal performance compared to heat-cured variants [1-3].

The observed reduction in water sorption can be attributed to the pressure-induced compression of the resin during the dough-to-set transition phase, which minimizes the microporosity that serves as a hydrophilic pathway for water ingress. In auto-polymerized systems, incomplete monomer conversion and entrapped air bubbles typically lead to higher porosity, exacerbating water sorption and subsequent hydrolytic degradation. The hydrostatic pressure, which is transmitted uniformly via the surrounding water bath, likely forces these bubbles to dissolve or redistribute, resulting in a denser microstructure. Kostić et al. [8] reported analogous reductions in water uptake for pressure-treated resins, linking it to a lower residual monomer content, which correlates with reduced polarity and hydrophilicity. These corroborative findings validate our results, suggesting that the custom pressure vessel offers a practical, low-cost alternative to vacuum mixing or microwave-assisted methods to achieve comparable outcomes in clinical settings.

Regarding flexural strength, the enhancement likely stems from the same densification process, where pressure eliminates stress concentrators, such as voids, thereby distributing the applied loads more evenly across the specimen. In three-point bending tests, materials with reduced internal defects exhibit higher fracture resistance because microcracks propagate less readily from flaws. This is consistent with the viscoelastic behavior of PMMA-based resins, where improved homogeneity under pressure enhances intermolecular forces [2,3]. Murakami et al. [7] reported higher flexural strength and elastic modulus for acrylic resins polymerized under a pressure of 500 MPa. Similarly, Babu et al. [9] reported that the R S tension clamping system led to a dimensionally stable acrylic resin. Consani et al. [10] reported less polymerization shrinkage with acrylic dentures polymerized using a hydraulic press, emphasizing the role of uniform polymerization in mitigating brittle failure. Thus, our results extend these observations by quantifying the benefits of a standardized orthodontic mold, implying that pressure treatment could yield more resilient appliances that are less prone to deformation under occlusal forces.

The lack of significant differences in polymerization shrinkage may reflect the limitations of volumetric measurement in capturing subtle pressure effects, as shrinkage in auto-polymerized resins is predominantly governed by monomer conversion rates rather than by external compression alone [2]. Although pressure theoretically constrains expansion, the short polymerization time (15-20 minutes) and moderate pressure levels may not sufficiently alter the intrinsic volumetric contraction (~6-8% for PMMA). Ono et al. [11] reported an increased dimensional accuracy of resin polymerized using a new injection-pressing polymerization pot.

Similarly, the surface hardness remained unchanged, likely because Vickers testing probes superficial layers, where polymerization completeness is primarily influenced by initiator diffusion rather than bulk pressure. A previous study has shown that hardness is insensitive to post-mixing pressures in acrylics as it correlates more with the degree of conversion at the surface, which our polishing protocol standardized across groups [12]. These non-significant outcomes indicate that pressure intervention selectively targets bulk properties, preserving ease of fabrication without necessitating adjustments for hardness-dependent clinical manipulations.

Color stability was unaffected in our study, suggesting that pressure does not alter chromophore formation or surface chemistry relevant to staining. Coffee staining primarily involves the adsorption of tannins onto the resin's porous network; thus, while reduced porosity might intuitively limit uptake, the ΔE values imply equivalent extrinsic discoloration potential [13]. This is substantiated by research from Gujjari et al. [14], who evaluated staining in conventionally cured acrylics and found no differences in ΔE after pigment exposure, attributing the stability to the inherent pigmentation resistance of PMMA. The staining solutions did not affect the flexural strength of PMMA, and the authors concluded that PMMA was resistant to damage from the staining solutions. In contrast, Narde et al. [15] reported inferior color stability and increased surface roughness for PMMA. Our blind assessment and Grubbs' outlier detection further ensured the robustness of this finding, highlighting the aesthetic neutrality of the treatment.

Clinical implications

From a clinical standpoint, these enhancements position the pressure vessel as a valuable adjunct in orthodontic and prosthetic practice, particularly in resource-limited settings. Reduced water sorption can minimize dimensional instability in intraoral environments, potentially extending the lifespan of removable appliances by curbing warpage and microbial adhesion associated with moisture retention. Improved flexural strength implies greater resistance to functional stresses, reducing fracture rates in high-load areas, such as denture bases or orthodontic retainers, which could lower remake frequencies and enhance patient satisfaction. Notably, the absence of adverse effects on shrinkage, hardness, or color stability facilitates seamless integration into workflows without additional training or aesthetic concerns, promoting material predictability for auto-polymerized resins, which are indispensable for provisional and custom fittings.

Limitations

Despite these insights, several limitations of this study warrant consideration. The in vitro design, while controlled, does not replicate dynamic oral conditions, such as enzymatic degradation, pH fluctuations, or masticatory cycling, potentially overestimating long-term durability. The sample size, although adequately powered, limits generalizability to larger cohorts or diverse patient demographics. Reliance on a single commercial resin restricts its applicability to other formulations, and the variability of custom pressure vessels introduces potential inconsistencies not seen in commercial systems. SEM analysis is qualitative, lacking quantitative porosity metrics such as image thresholding, which could provide deeper mechanistic insights. Future studies should incorporate fatigue testing, biocompatibility assays, and multicenter trials to validate the clinical translation.

Additionally, minor procedural factors such as operator-dependent packing pressure and variations arising from the monomer-to-polymer ratio when measured by volume versus weight may have influenced the consistency of specimen preparation. The pressure range employed in this study (2.2-3.0 bar) is comparatively lower than that reported in high-pressure polymerization protocols, thereby limiting the generalizability of the observed effects. Furthermore, the use of lukewarm water during curing could have introduced mild thermal influences on polymerization kinetics. Finally, as no residual monomer quantification or chemical conversion analysis was performed, mechanistic interpretations regarding improved polymer conversion should be viewed as speculative and confirmed in future research.

## Conclusions

This study demonstrated that pressure vessel treatment during the polymerization of auto-polymerized acrylic resins significantly enhanced their mechanical and physical properties, notably reducing water sorption and increasing flexural strength, without altering polymerization shrinkage, surface hardness, or color stability. These improvements suggest that the pressure-curing method optimizes the resin matrix, making it a promising technique for fabricating more durable dental appliances, such as orthodontic retainers and denture bases. The findings support its potential integration into clinical practice, particularly in resource-limited settings, to improve material performance without compromising aesthetics. Further in vivo studies are required to validate its long-term clinical efficacy and broader applicability.
